# Supplementary material for: International and regional spread of carbapenem-resistant Klebsiella pneumoniae in Europe
Source: Nat Commun. 2024 Jun 14;15:5092. doi: 10.1038/s41467-024-49349-z (PMC11178878; doi:10.1038/s41467-024-49349-z)
Supplement: Supplementary file 1 — Supplementary Information [file 41467_2024_49349_MOESM1_ESM.pdf]

Supplementary Table 1: Characteristics of CRKP isolates EuSCAPE only recovered in Southern European countries

Clonal lineages	Total number of isolates (% of total)	Countries with more than 10 isolates	Number of different countries	STs with more than 10 isolates	Carbapenemase gene variants and number of isolates				No carbapenemase found
					Class A	Class B	Class D	Two carbapenemases	
ST258/512	166 (31%)	Italy (n=134)<br>Greece (n=27)	4	ST512 (n=116)<br>ST258 (n=50)	bla_{KPC-2} (n=32)<br>bla_{KPC-3} (n=133)<br>bla_{KPC-12} (n=1)				
ST11/437/340	89 (17%)	Croatia (n=31)<br>Greece (n=14)<br>Serbia (n=24)<br>Spain (n=13)	7	ST11 (n=33)<br>ST437(n=38)<br>ST340 (n=18)	bla_{KPC-2} (n=5)	bla_{NDM-1} (n=28)<br>bla_{VIM-1} (n=2)	bla_{OXA-48} (n=12)	3	45
ST101	83 (16%)	Serbia (n=26)<br>Romania (n=33)<br>Türkiye (n=15)	7	ST101 (n=83)	bla_{KPC-2} (n=4)<br>bla_{KPC-3} (n=1)	bla_{NDM-1} (n=9)	bla_{OXA-48} (n=62)	5	12
ST307	10(2%)		4	ST307 (n=10)	bla_{KPC-2} (n=1)<br>bla_{KPC-3} (n=2)	bla_{NDM-1} (n=1)	bla_{OXA-48} (n=3)		1
ST15	53 (10%)	Spain (n=24)<br>Romania (n=12)	5	ST15 (n=50)	bla_{KPC-2} (n=1)<br>bla_{KPC-3} (n=1)<br>bla_{KPC-12} (n=3)	bla_{NDM-1} (n=1)<br>bla_{VIM-1} (n=7)	bla_{OXA-48} (n=35)	1	6
ST147	25 (5%)		6	ST147 (n=22)	bla_{KPC-2} (n=6)	bla_{NDM-1} (n=2)<br>bla_{VIM-1} (n=8)	bla_{OXA-48} (n=11)	3	1
ST14	3		1				bla_{OXA-48} (n=3)		
ST405	13	Spain (n=10)	2	ST405 (n=13)			bla_{OXA-48} (n=11)		2
ST37	4		3				bla_{OXA-48} (n=4)		
ST45	4		3				bla_{OXA-48} (n=3)		1
ST17	5		2				bla_{OXA-48} (n=5)		
Other	71	Türkiye (n=36)	8	ST274 (n=11)	bla_{KPC-2} (n=1)<br>bla_{KPC-3} (n=3)	bla_{NDM-1} (n=19)<br>bla_{VIM-1} (n=13)	bla_{OXA-48} (n=27)	4	8

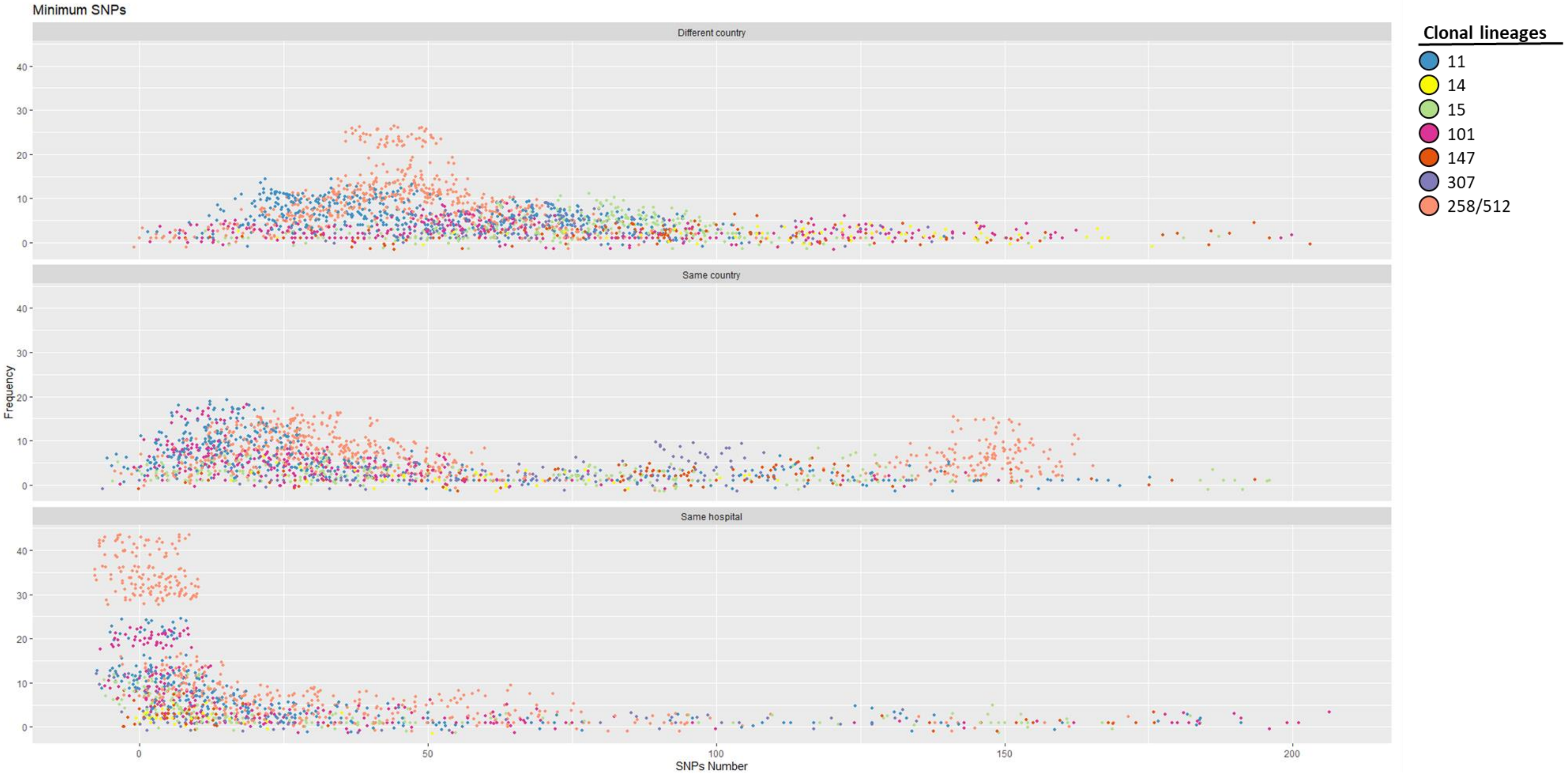

Supplementary Figure 1: Determination of minimum SNP distribution for isolates from the same hospital (a), different hospitals but same country (b), and different countries (c). Colours depict different clonal lineages, with ST258 as a comparator from the EuSCAPE analysis but including the EURECA isolates.

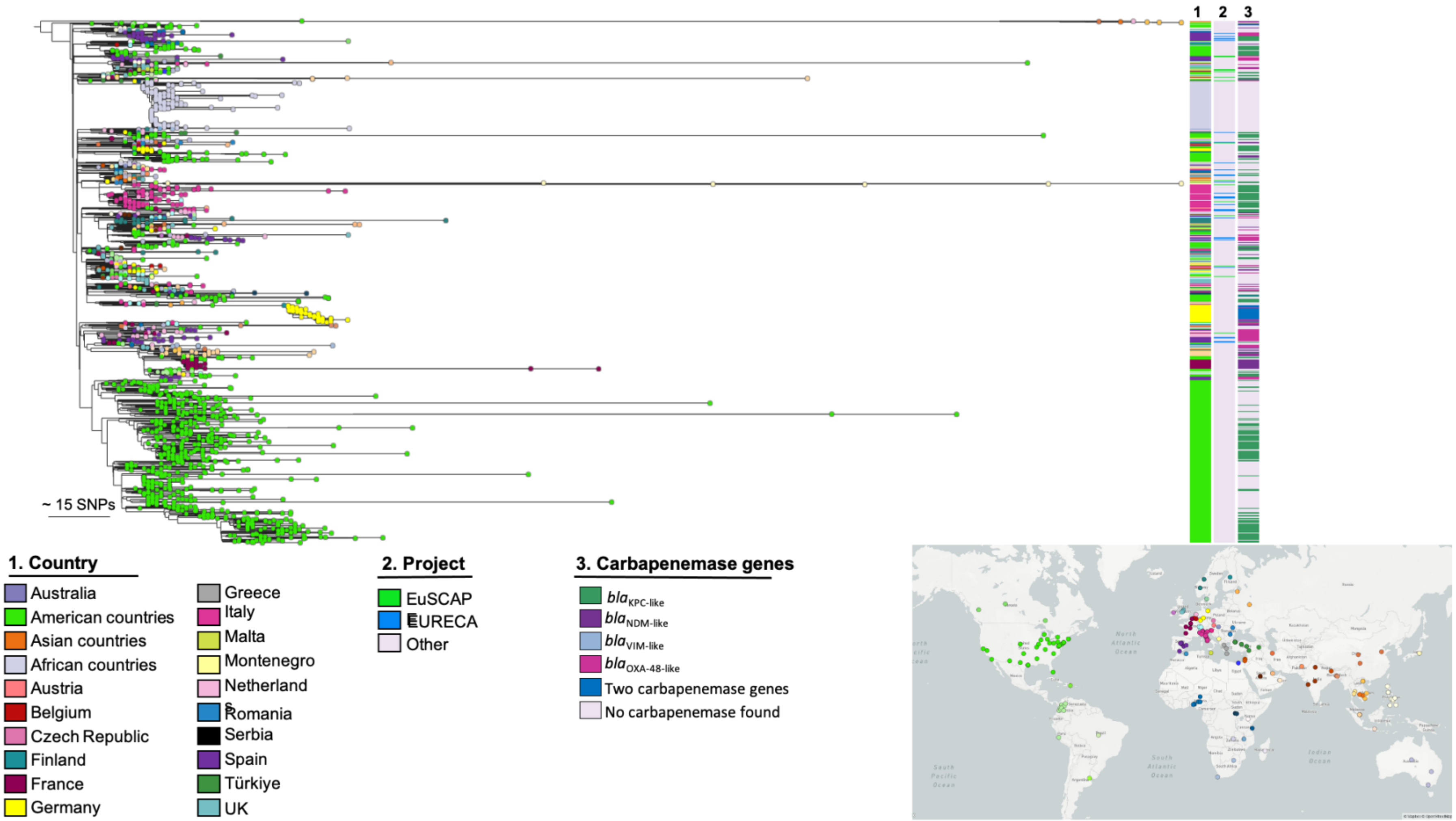

Supplementary Figure 2: A phylogenetic tree of *Kp* ST307 genomes available publicly from around the world. Columns shows 1) country of isolation, 2) projects, 3) type of carbapenemase gene. Tree tip colours correspond to country. Inset map shows location of isolates in countries. Map has been created using OpenStreetMap is included here under CC BY-SA 2.0 licence (https://www.openstreetmap.org/copyright). Also available in microreact: https://microreact.org/project/gB8xB89kcw4B6jFztEy1wq-st307globalcollectioneurecaproject

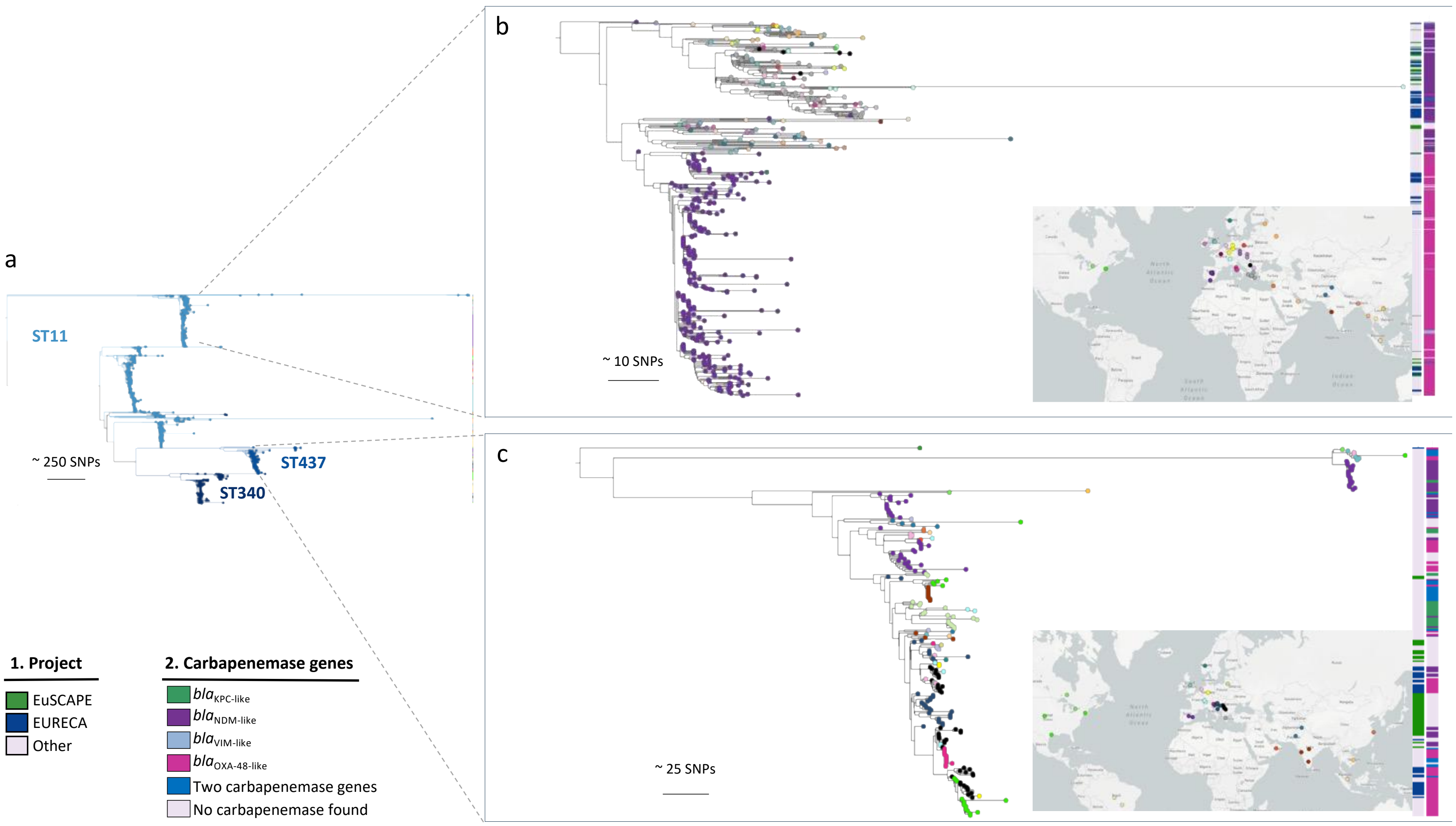

Supplementary Figure 3: Phylogenetic analysis of ST11 and variants ST437 and ST340 global collections. a) Isolates grouped by ST; b) clade of ST11 shows in detail the dissemination of one clone in Spain (purple) and other countries visible in a map; c) clade of ST437 shows in detail the dissemination of one clone in Serbia (black) Italy (pink) Crotia (petro blue) and other countries visible in a map. In the column 1 isolates of EURECA (blue) and EuSCAPE (green) isolates differ from the other projects, column 2 different colours to each carbapenemase type**.** Map has been created using OpenStreetMap is included here under CC BY-SA 2.0 licence (https://www.openstreetmap.org/copyright). Available in microreact: https://microreact.org/project/b2kcRm7LKhVMUqNn3jrXcw-globalcollectionst11437340eurecaproject

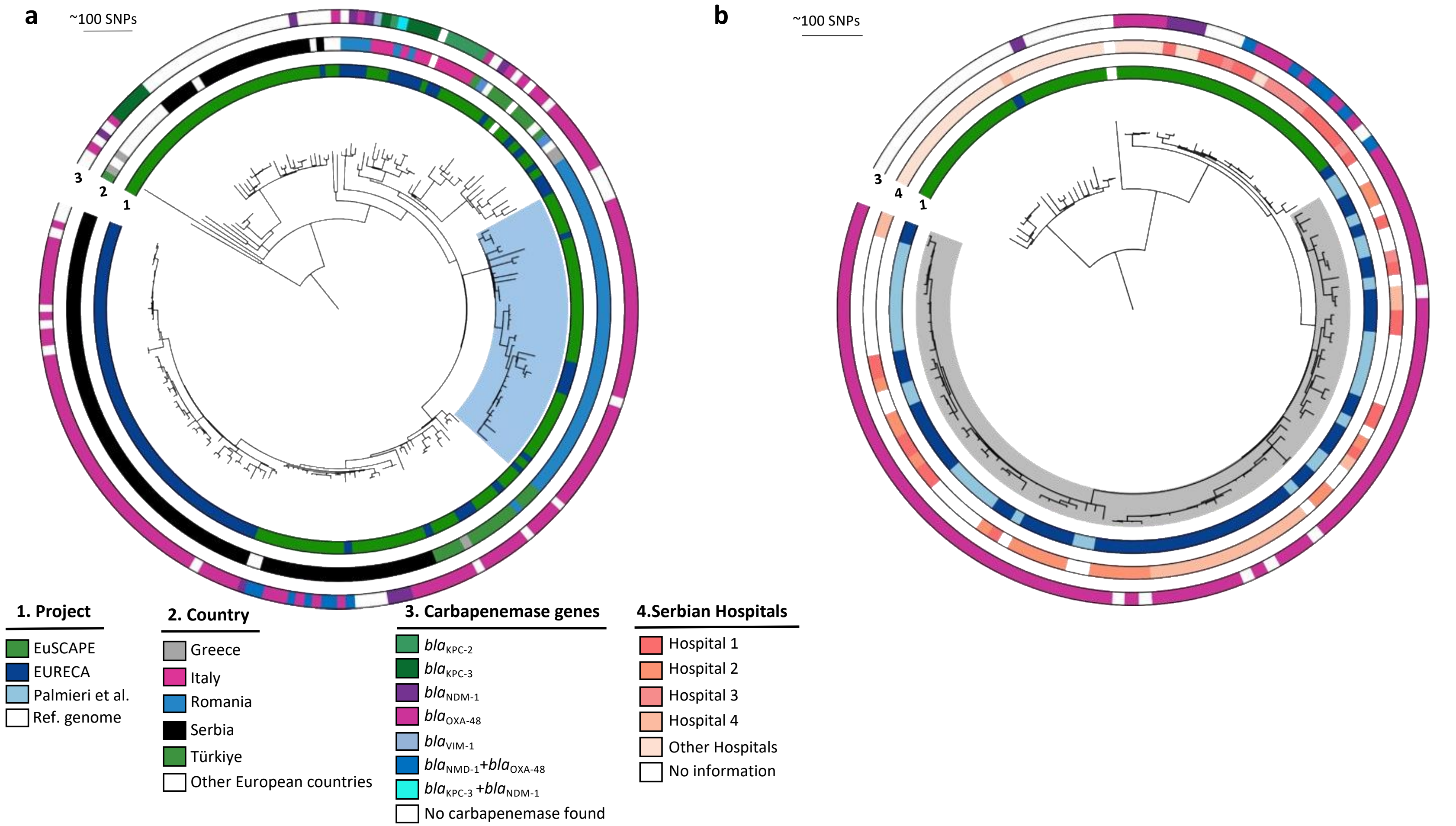

Supplementary Figure 4: Phylogenetic analysis of ST101. a. Phylogenetic tree of 213 isolates from EURECA and EuSCAPE survey. Reference genome Kp_Goe_33208 (CP018447). Inner ring: project (1), middle ring: geographic origin (2), outer ring: carbapememase genes (3). Blue box depicts Romanian clade with *bla*$_{\text{OXA-48}}$. **b.** 135 isolates ST101 from Serbia recovered during EuSCAPE, EURECA survey and Palmieri et al study (Palmieri et al., 2020). The rings show from inside to outside the project (1), different colours representing different hospitals in Serbia (4), and carbapenemase genes (3). Grey box highlights the expansion including the EURECA and Palmieri isolates.

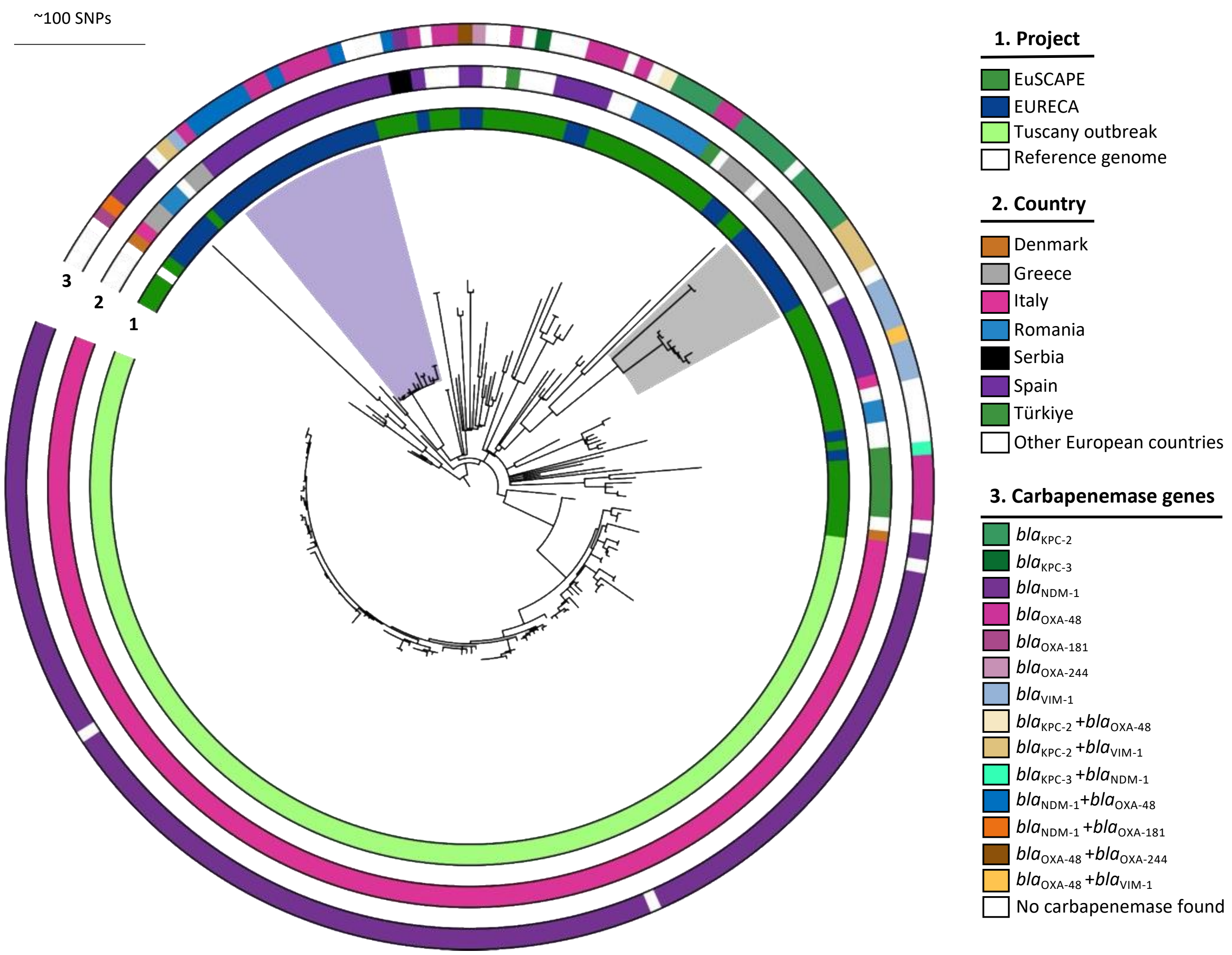

Supplementary Figure 5: Phylogenetic analysis of ST147. Phylogenetic tree of 212 isolates from EURECA, EuSCAPE survey and an outbreak in Tuscany Italy (Martin et al., 2021). Referene genome HKP0064 (JACTAR01). The rings show the project (1), geographic origin (2), and carbapenemase genes (3). The purple and grey boxes highlight particular clades of interest from Spain and Greece, respectively.

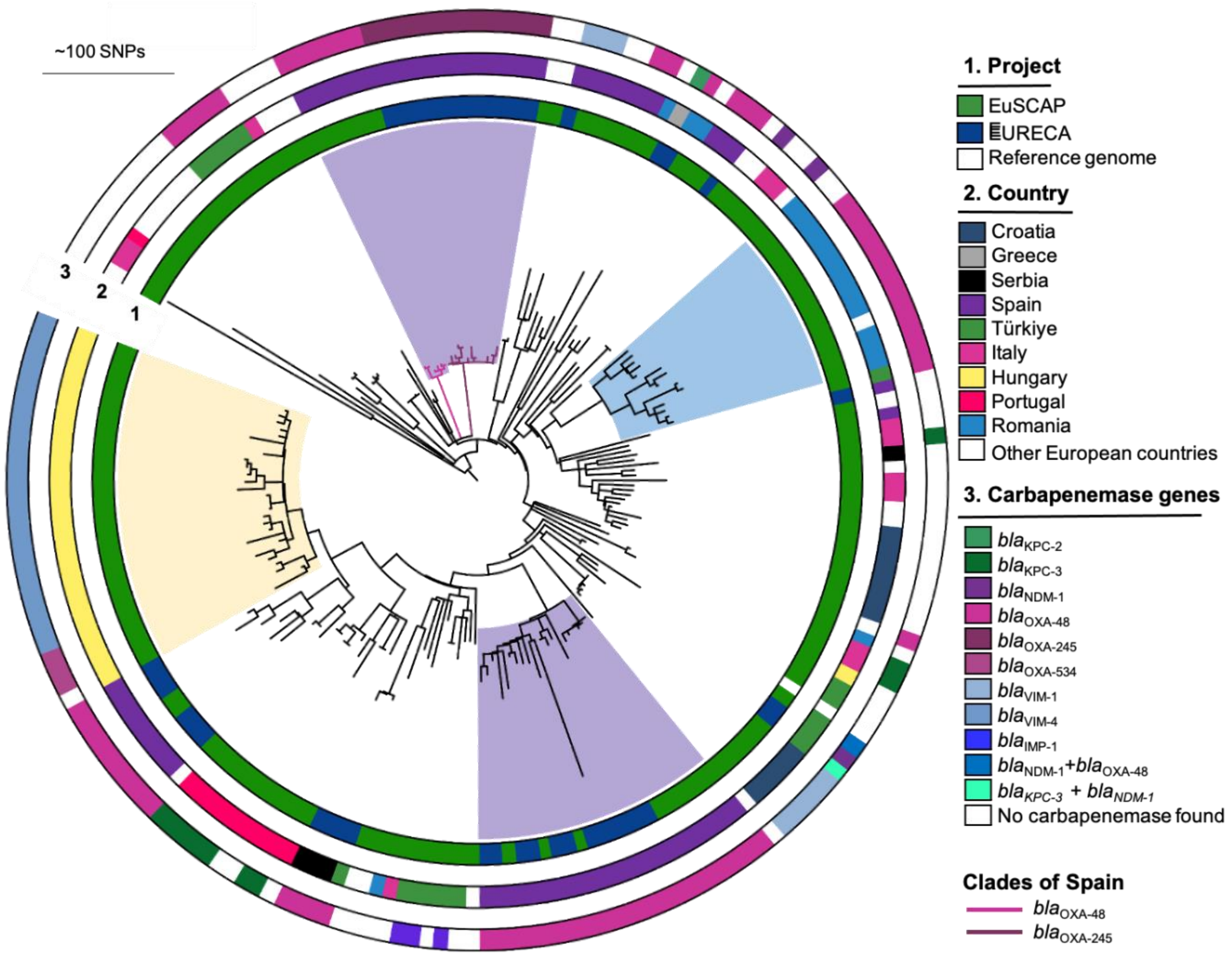

Supplementary Figure 6: Phylogenetic analysis of ST15. Phylogenetic reconstruction of 189 isolates from EURECA and EuSCAPE survey. Reference genome P35 (CP053041). The rings show the project (1), geographic origin (2), SNP distance (3), and carbapenemase genes (4).

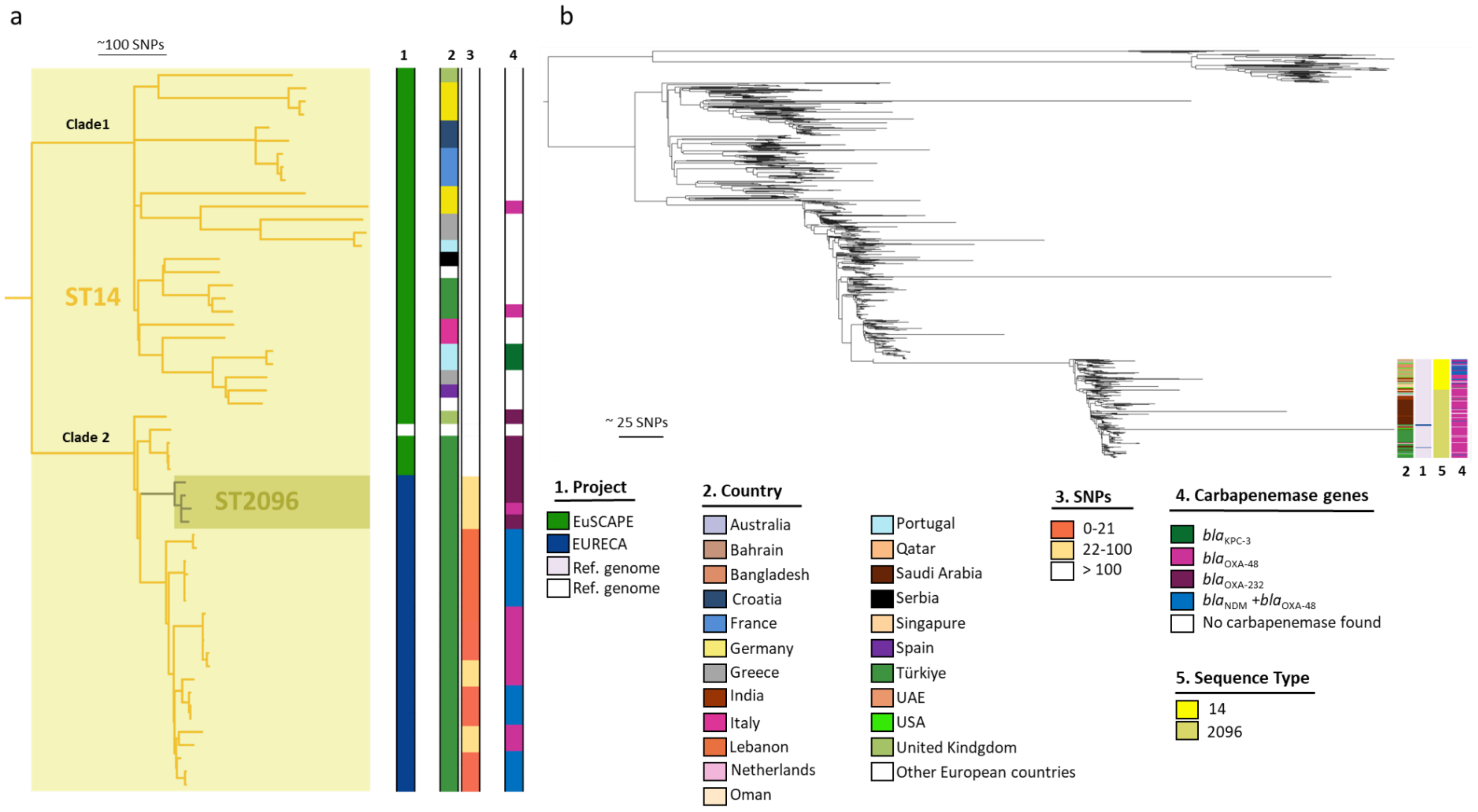

Supplementary Figure 7: Phylogenetic analysis of ST14. Phylogenetic tree of 54 isolates from EURECA and EuSCAPE survey. Reference genome KPN528 (CP020856). The rings show the project (1), geographic origin (2), SNP distance (3), and carbapenemase genes (4). B) subset of isolates derived from https://microreact.org/project/3mPo67JrFnL6Xt3cVSVrR1-st142096lineageglobalcollectionproject
