# Supplementary material for: International and regional spread of carbapenem-resistant Klebsiella pneumoniae in Europe
Source: Nat Commun. 2024 Jun 14;15:5092. doi: 10.1038/s41467-024-49349-z (PMC11178878; doi:10.1038/s41467-024-49349-z)
Supplement: Supplementary file 3 — Description of Additional Supplementary Files [file 41467_2024_49349_MOESM3_ESM.pdf]

Description of Additional Supplementary Files

Supplementary Data 1: Sequencing information and statistics, metadata, and derived genotypes of isolates sequenced in this study.

Supplementary Data 2: Kleborate output including virulence factors and capsule types for isolates in this study.

Supplementary Data 3: Additional datasets and available metadata used in the analyses.
